# Strangulated recurrent rectal prolapse after a Delorme intervention, a case report

**DOI:** 10.1002/ccr3.2087

**Published:** 2019-03-06

**Authors:** Nadim Malibary, Cecile Brigand

**Affiliations:** ^1^ Department of Surgery King Abdulaziz University Jeddah Saudi Arabia; ^2^ Department of General and Digestive Surgery Hautepierre University Hospital Strasbourg France

**Keywords:** Delorme, prolapse complications, rectal prolapse, strangulation

## Abstract

A surgeon must be aware of indications of rectal prolapse surgery with proper preoperative evaluation and have the ability to recognize complications. Sigmoid redundancy might lead to early recurrence, which itself could complicate into strangulation. Complications per say might require more drastic measures, as Hartmann's procedure in this case.

## 1 INTRODUCTION

Rectal prolapse is defined as the protrusion of the rectum through the anus. Its incidence is most common among children and the elderly.^1^

More than 100 techniques have been described in the literature, based on two approaches: transabdominal and transperineal procedures. The most common transabdominal surgeries are ventral rectopexy with a synthetic mesh, either laparoscopically or by laparotomy. The perianal approaches include the Delorme and Altemeier procedures at 50%‐60% of all transperineal interventions. No formal consensus or guidelines have been described to support a single gold standard intervention.^2^

Between the abdominal and perineal approaches and with the emergence of minimally invasive surgery, various studies have shown that the perineal approach is no longer restricted, but remains preferred for surgically unfit patients. Recurrence rates and morbidities are almost equal.^2^, ^3^, ^4^

One randomized clinical trial called “DeloRes” compared Delorme and rectopexy; however, results are pending.^5^

## 2 CASE REPORT

This is the case of an 85‐year‐old female who presented with a strangulated recurrent rectal prolapse less than a month after a Delorme surgical repair. During the first encounter with her surgeon for her rectal prolapse, she was offered the Delorme intervention to avoid any surgical stress due to her associated Takotsubo syndrome that was diagnosed early in 2016. No preoperative radiological examinations were performed, and the patient was operated on in December 2016.

Shortly thereafter, she was referred to our department by her family physician for a recurrent prolapse of 10 cm. At our clinic, the prolapse was reducible and the anal tone was weak with barely any tone while squeezing. The patient was hardly passing stool since the recurrence of the prolapse.

A corrective mesh ventropexy (D'Hoore) surgery was proposed, and the patient gave consent.

**Figure 1:**
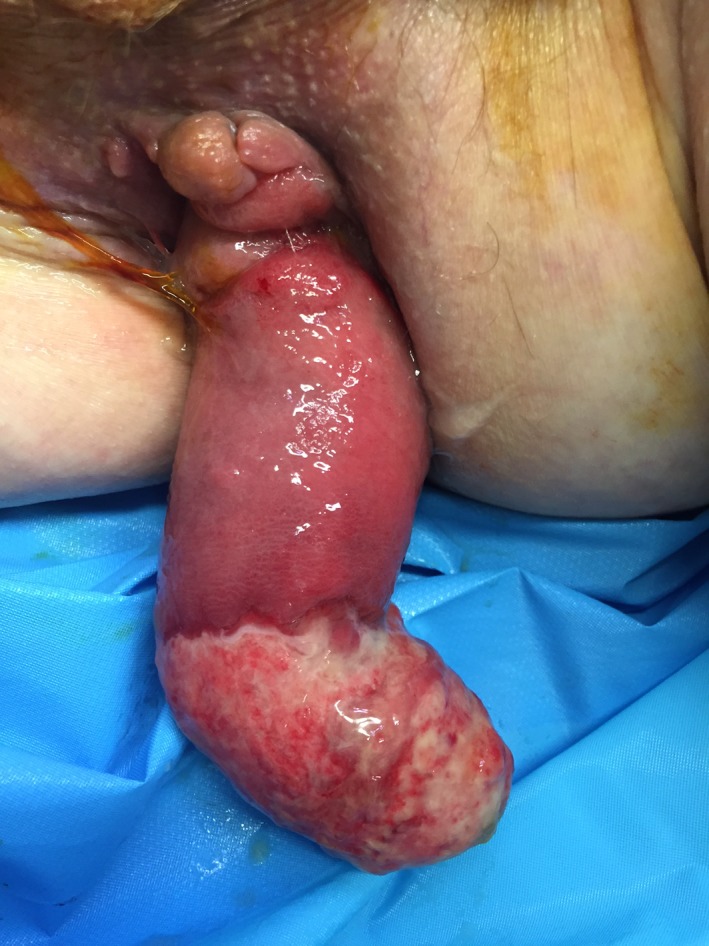
The incarcerated recurrent rectal prolapse. The suture line is visible and intact. Necrosis on the tip

On admission day, the prolapse was even more exteriorized at approximately 20 cm, with circumferential necrosis of its extremity of approximately 10 cm. The suture line of the previous operation was visible and intact (Figure 1). The patient complained of abdominal discomfort and constipation. The prolapse was irreducible, and the patient was in a subocclusive state.

Because of our clinical findings, and after combining the weak anal tone and the necrosis, we decided to proceed with a Hartmann intervention after obtaining consent from the patient, primarily to avoid fecal incontinence for a better quality of life.

**Figure 2:**
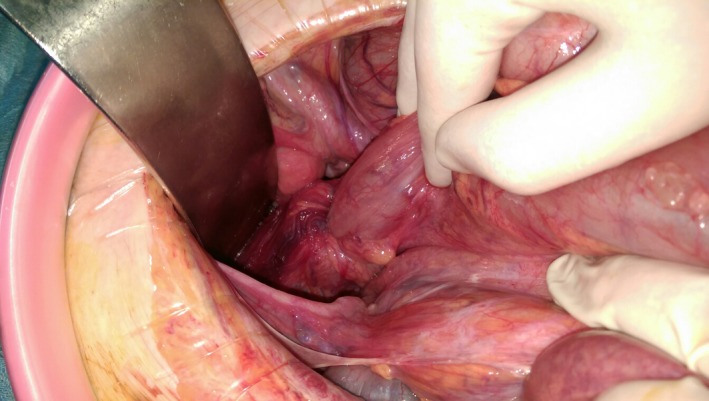
Intraoperative view of the colorectal intussusception and the patch of necrosis

An infraumbilical laparotomy was performed. The patient was in obstruction status caused by a colorectal intussusception, due to a very redundant sigmoid (Figure 2).

A manual reduction of the prolapse was possible by pushing from the outside and pulling on the sigmoid from the inside of the abdominal cavity. Upon examining the rectum, we found patches of necrosis and a sealed perforation.

We performed a Hartmann procedure leaving a very short rectal stump, with a left terminal colostomy.

The postoperative period was short and uneventful.

The histopathology report showed severe ulcerations and acute inflammatory response. No other pathology was detected.

The patient was seen 1‐ and 6‐month postoperatively. She was doing fine and able to handle her colostomy with the help of her son. No residual prolapse was seen, and the digital rectal exam showed the very weak sphincter tone and a 3‐cm rectal stump.

## 3 DISCUSSION

The Delorme procedure entails a mucosal sleeve resection, proximal to the dentate line, with longitudinal rectal muscular plication.^3^, ^4^

It has a higher recurrence rate than does the Altemeier procedure, probably because we do not enter the peritoneal cavity. According to the literature, recurrence ranges between 7% and 27%,^3^, ^5^ similar to that of the abdominal approaches.^2^

Warwick et al^2^ developed a combined Delorme‐Thiersch (anal encirclement) procedure to reduce recurrence from 21% to 8%, with no increases in the complication rate.

Recurrence is most likely due to persistent postoperative constipation, concomitant pelvic floor weakness, or a redundant colon,^6^, ^7^ the latter two being the cause in our case, especially because the redundant colon cannot be detected clinically.

Many possibilities exist to treat recurrences. One systemic review showed the success of repeated Delorme or Altemeier, an abdominal approach such as ventropexy, and even a hybrid double approach.^7^, ^8^ One recent article has proposed the Delorme intervention as the first intervention for treating recurrences.^6^

Incarcerated or strangulated rectal prolapse is very rare (2%‐4%), and necrosis of the stump is even rarer.^1^ No statistics were found for recurrent cases. Although most common in the elderly, incarceration can also occur in younger patients.^1^

The most common approach for strangulation is Altemeier.^9^

To our knowledge, this is the first case describing a necrotic strangulated recurrent prolapse after a Delorme procedure. Two case reports were found describing the phenomenon after an Altemeier operation.^8^, ^10^

## 4 CONCLUSION

Strangulation of a rectal prolapse is very rare, even more so after corrective surgery. This case is presented to show another possible presentation of recurrent rectal prolapse, which to our knowledge has not been described previously in the literature.

## CONFLICT OF INTEREST

None declared.

## AUTHOR CONTRIBUTION

NM: studied the concept, and designed and wrote the paper. CB: supervised and critically revised the paper.
